# Is the Planus Foot Type Associated With First Ray Hypermobility?

**DOI:** 10.1177/24730114221081545

**Published:** 2022-03-04

**Authors:** Oliver J. Morgan, Rajshree Hillstrom, Robert Turner, Jonathan Day, Ibadet Thaqi, Kristin Caolo, Scott Ellis, Jonathan T. Deland, Howard J. Hillstrom

**Affiliations:** 1 Medical Engineering Research Group, Faculty of Science and Engineering, Anglia Ruskin University, Chelmsford, Essex, United Kingdom; 2 Biomed Consulting, Inc, New York, NY, USA; 3 Leon Root, MD Motion Analysis Laboratory, Hospital for Special Surgery, New York, NY, USA; 4 Department of Foot and Ankle Surgery, Hospital for Special Surgery, New York, NY, USA

**Keywords:** foot, foot type, first ray mobility, first ray hypermobility, first metatarsophalangeal joint flexibility, biomechanics

## Abstract

**Background:**

Many foot pathologies have been associated with foot type. However, the association of first ray hypermobility remains enigmatic. The purpose of this study was to investigate first ray hypermobility among participants with planus and rectus foot types and its influence on static measures of foot structure.

**Methods:**

Twenty asymptomatic participants with planus (n = 23 feet) and rectus (n = 17 feet) foot types were enrolled. Several parameters of static foot structure (arch height index, arch height flexibility, first metatarsophalangeal joint flexibility, and first ray mobility) were measured. Participants were further stratified into groups with nonhypermobile (n = 26 feet) and hypermobile (n = 14 feet) first rays. First ray mobility **≥**8 mm was used to define “first ray hypermobility”. Generalized estimating equations, best-fit regression lines, and stepwise linear regression were used to identify significant differences and predictors between the study variables

**Results:**

Overall, 86% of subjects categorized with first ray hypermobility exhibited a planus foot type. Arch height flexibility, weightbearing first ray mobility, and first metatarsophalangeal joint flexibility showed no significant between-group differences. However, weightbearing ray mobility and first metatarsophalangeal joint laxity were associated with partial weightbearing first ray mobility, accounting for 38% of the model variance.

**Conclusion:**

The planus foot type was found to be associated with first ray hypermobility. Furthermore, weightbearing first ray mobility and first metatarsophalangeal joint laxity were predictive of partial weightbearing first ray mobility, demonstrating an interaction between the translation and rotational mechanics of the first ray.

**Clinical Relevance:**

Association of first ray hypermobility with foot type and first metatarsophalangeal joint flexibility may help understand the sequela to symptomatic pathologies of the foot.

## Introduction

The first ray is a single foot segment comprised of the hallux, first metatarsal, and medial cuneiform.^19,20^ Mobility of this segment may be quantified by superior translation when subjected to a dorsally directed load. *Hypermobility*^17,35,43^ is a term that describes excessive mobility of the first ray beyond what is considered “normal.”^11,17,44,51^ Clinical definitions can vary based on differing case definitions and modes of assessment.^11,15,17,26,29,36,44,49,51^ However, superior translation of the first ray ≥8 mm has been used to define hypermobility.^17^ The first ray can be affected by common orthopaedic disorders of the foot, including hallux rigidus^32,33,42^ and hallux valgus.^2,9,25,38^ Many possible causative factors have been rejected owing to a lack of convincing evidence^4,5,42,44,50^ and so the role of first ray hypermobility in abnormal and potentially harmful pedal mechanics remains enigmatic.

Many pathologies of the foot may be biomechanical in origin and have been associated with foot type,^27,28,30,37,47,48^ which can be divided into 3 distinct classifications: planus (a low arch with a valgus calcaneus and/or supinated forefoot), rectus (a moderate arch with a neutral calcaneus and forefoot), and cavus (a high arch with varus calcaneus and/or pronated forefoot).^28^ These structural references describe common morphologic and structural variations among the general population. It is generally accepted that foot function and structure are related to one another and that functional variations exist between these 3 classifications.^6,7,22,28^ Subjects with pes planus have demonstrated greater odds of developing foot injuries,^24^ increased first metatarsophalangeal (MTP) joint flexibility,^39^ greater plantar loading of the hallux and second metatarsal,^6,22^ and a higher odds ratio of hallux rigidus.^32^ Several investigators have indirectly^22,28^ or anecdotally^35,43^ linked first ray hypermobility to the planus foot type as a mechanism of aberrant pedal biomechanics. However, there is limited research of a link between foot type and first ray mobility.^11,13^

The present investigational team developed and previously published the reliability^34^ of a device (MAP1^st^) to address the limitations of prior methods for measuring first ray mobility. The current research objectives were to assess first ray hypermobility in asymptomatic subjects with planus and rectus feet, in addition to determining if measures of static foot structure (ie, arch height index, arch height flexibility, and first MTP joint flexibility) were related to first ray mobility. To test the research objectives, 2 hypotheses were established: (1) first ray hypermobility will demonstrate an association to individuals with a planus foot type; (2) first ray mobility will be related to first MTP joint flexibility.

## Methods

The study included 23 asymptomatic planus feet and 17 asymptomatic rectus feet for a total of 20 participants (N = 40 feet). All procedures were approved by the Hospital for Special Surgery Institutional Review Board. Testing was performed at the Leon Root, MD, Motion Analysis Laboratory at HSS, where each subject was consented before testing. Flyers were distributed and an announcement was made for subject recruitment at the institution. Participants that met the inclusion/exclusion criteria were enrolled in the study (Table 1). Participant characteristics are shown in Table 2. One independent examiner performed the measurements. One subject with pes rectus was excluded owing to presentation of generalized joint hypermobility.^45^ This participant demonstrated hyperextension of the little finger, apposition of the thumb to the flexor aspect of the forearm, hyperextension of the elbow, and forward flexion of the trunk so that palms of the hands rested on the floor.^3^ No other participants demonstrated generalized joint hypermobility. All other subjects were categorized according to their foot type (ie, planus or rectus) using arch height index,^9,22^ and further assessed for first ray mobility, arch height flexibility, and parameters of first MTP joint flexibility.

**Table 1.** Inclusion/Exclusion Criteria for Subjects Enrolled in the Study.

Inclusion	Exclusion
• Healthy adults aged >21 y	• Individuals without the capacity to consent and/or understand procedures of the study
• No substantial pain within the lower extremity that could affect ability to walk	• Hallux valgus, hallux rigidus, rheumatoid arthritis, osteoporosis, or any other degenerative disease involving the lower limb
• Male or female	• Any disease or pathology affecting one’s ability to walk independently or limb length discrepancy >1 cm
• Planus: AHI_standing_ <0.345	• Generalized joint hypermobility
• Rectus: 0.345≤ AHI_standing_ ≤0.37	• Cavus: AHI_standing_ >0.37

Abbreviation: AHI, arch height index.

**Table 2.** Participant Characteristics by Foot Type.

	Planus (n = 23 feet)		Rectus (n = 17 feet)		GEE Results	
Parameters	Mean	SD	Mean	SD	χ^2^	*P* Value
Age, y	33	3	33	3	1.711	.191
Height, cm	176	2	176	2	0.050	.824
Weight, kg	78	3	78	3	0.000	.988
BMI	25	1	25	1	0.041	.840

Abbreviations: BMI, body mass index; GEE, generalized estimating equation.

## Foot Type

**Figure 1.**
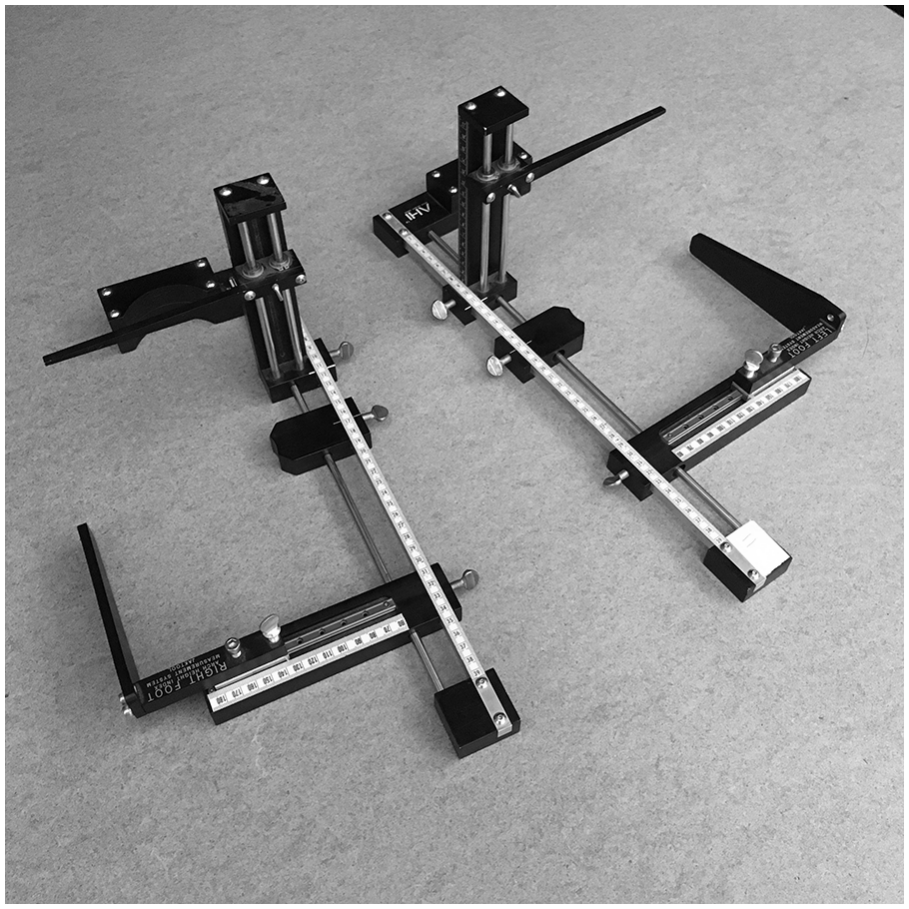
Photograph of the arch height index system. During testing, a subject's left and right feet were placed in the corresponding devices, assessed for arch height index, and categorized into planus or rectus foot types.

Arch height index can reliably distinguish planus and rectus foot types in healthy individuals, according to thresholds previously established.^9,22^ Each of the subject’s feet were positioned in the arch height index device, with the most anterior bar set to maximum foot length (Figure 1). A small adjustable cup was positioned at the first MTP joint to denote truncated foot length (TFL) and a vertical bar, which was positioned at one-half of foot length, was lowered on the dorsal aspect of the foot to measure arch-height. Linear rulers, scaled in centimeters, were used to visually measure each parameter. The graticule was viewed by the rater with their aiming eye, in a perpendicular orientation, to avoid parallax error. Arch height index was defined as the dorsal arch height at one-half of foot length, normalized by TFL, while standing (AHI_standing_).

## First Ray Mobility

**Figure 2.**
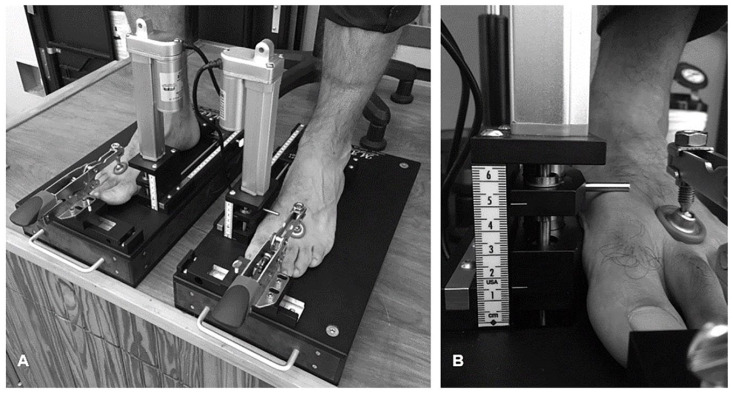
Photographs of MAP1^st^: (A) left and right device and (B) metatarsal head height with 50 N applied to measure first ray mobility. The upper indicator (located at the dorsal aspect of the first metatarsal head) can be seen to displace by 14 mm from its initial position to its loaded position.

MAP1^st^ was used to quantify first ray mobility. The device was previously found to be reliable with a standard error of measurement ranging from 0.3 to 0.4 mm.^34^ Prior to measurements of first ray mobility, 10 successive 25-N loading cycles were used to control the recent strain history of the soft tissues.^52^ First ray mobility, using MAP1^st^, was measured while the subjects were seated (ie, lower extremity positioned in 90 degrees of hip and knee flexion) and standing (ie, hip-knee-ankle in 0 degrees alignment) for partial- and full-weightbearing assessments, respectively (Figure 2). The foot was placed in resting calcaneal stance position (RCSP) and the ankle in a neutral alignment for both partial- and full-weightbearing measurements.^18^ Resting calcaneal stance position may be defined as a relaxed position of the foot with the medial longitudinal arch in its natural weightbearing alignment.^22^ Measurements were calculated based on linear displacement of the first ray for measurements of FRM (in millimeters). The measurement of first ray mobility was determined from linear displacement of the dorsal first metatarsal head after 50 N of load. To perform the measurements, an independent recorder viewed MAP1^st^’s graticule with their aiming eye, in a perpendicular orientation, to avoid parallax error and recorded dorsal displacement.

## Arch Height Flexibility

The arch height of an individual can be calculated either in sitting (ie, partial weightbearing) or standing (ie, weightbearing) positions for partial- and full-weightbearing assessments, respectively. Arch height flexibility (millimeters/kilonewtons) is a measure of the change in arch height between the sitting and standing conditions (Figure 1). The measurement is normalized to the change in load (estimated to be 40% of bodyweight).^12,22^ Calculation of arch height flexibility is made with the following formula:

(1) $$\mathrm{AHF}\left(\frac{\mathrm{mm}}{\mathrm{kN}}\right)=\frac{\left|AH_{standing}\left(mm\right)-AH_{sitting}\left(mm\right)\right|}{0.4\times \mathrm{bodyweight}\left(\mathrm{kN}\right)}x100,$$

In the absence of a limb length discrepancy, approximately 50% of body weight will be borne by each limb during standing. The percentage of bodyweight (BW) while sitting may be estimated from anthropometric data. Bodyweight of the foot, shank, and thigh was measured at 1.4%, 4.6%, and 10%, respectively.^12^ When seated, the weight of the foot, shank, and 40% of thigh contribute to the vertical force of the lower limb. The 40% of the thigh weight is estimated as follows: When seated on a stool, with the hip and knee at 90 degrees, 20% of the thigh contacts the seat. The remaining 80% of the thigh weight is shared between the seat and the shank. Therefore, 40% of the thigh weight (4% BW) will contribute to the vertical load while seated and, thus, the total vertical load while sitting is 1.4% + 4.6% + 4% = 10% BW. The difference between 50% BW when standing and 10% BW while sitting is 40% or 0.4, as calculated for arch height flexibility.

## First Metatarsophalangeal Joint Flexibility

**Figure 3.**
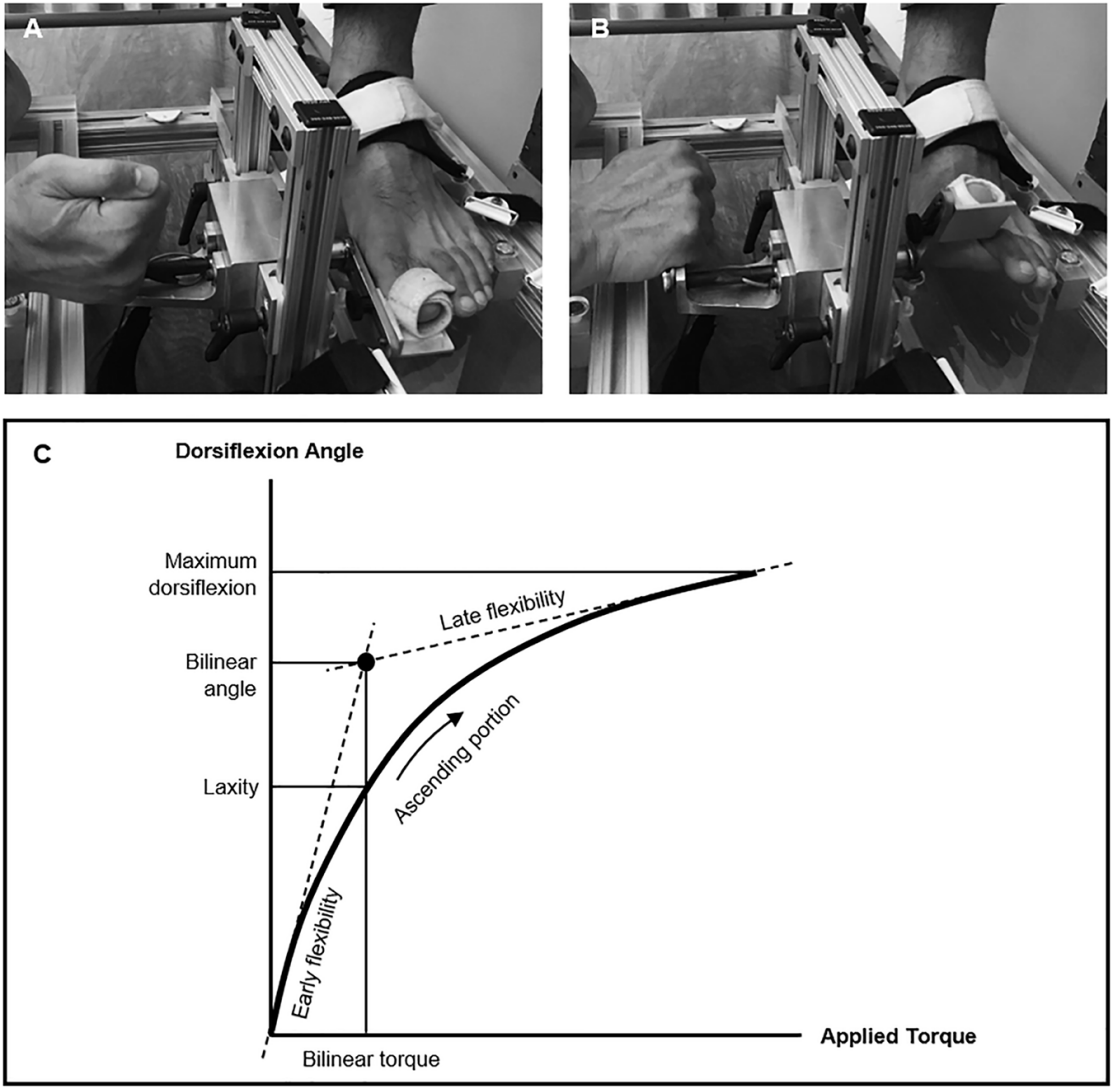
(A) the subject’s foot is placed in the test-rig, where their hallux is strapped to a pivot mechanism and the mid- and rear-foot are immobilized by Velcro straps; (B) the tester applies a torque to dorsiflex the hallux and measure the residual torque using a transducer integrated into the pivot mechanism. (C) Illustrated diagram of the first metatarsophalangeal joint flexibility curve. The intersection of the early and late flexibility slope lines is denoted by a reference point. The coordinates for this point, at the *x* and *y* axes, define the bilinear torque and bilinear angle, respectively. The bilinear angle is the on the *y* axis at which the value for normal bilinear torque intersects the flexibility curve. Laxity is the amount of angular rotation observed for a standardized amount of applied torque.

The first MTP joint flexibility test-rig (Figure 3A, B) was previously found to be reliable.^10,39^ Standard error of measurement values have ranged from 1.52 degrees/Ncm to 3.49 degrees/Ncm.^39^ The left and right feet of each subject were tested to provide bilateral assessments of first MTP joint flexibility. During testing, each patient was seated in a chair with their knees flexed to 90 degrees and thighs parallel to the floor and their ankle in a neutral alignment.^18^ The flexibility test-rig was connected to a laptop running DAQami software (Measurement Computing Corporation), which recorded the voltage signals for first MTP joint torque (Ncm) and dorsiflexion (degrees). Before recording, the first MTP joint of each foot was cyclically loaded 10 times to control for the soft tissue’s recent strain history.^52^

## Statistics

The normality of data distribution was assessed using Shapiro-Wilk test. Generalized estimating equation (GEE) analyses were used to compare age, height, weight, and BMI of participants by foot type. Descriptive statistics (frequency, mean, and SD) were computed for each group. Participants were then stratified into nonhypermobile (<8 mm) and hypermobile (≥8 mm) groups. First ray mobility was considered the primary dependent variable in the present study. GEE analyses were performed across foot type and first ray mobility for parameters of foot structure. A *P* <.05 suggested a significant difference. Cohen’s *d* was computed to assess the effect size of the mean differences. Best-fit regression lines were created and a stepwise linear regression model was also employed to determine predictors of first ray mobility. Significance levels for inclusion and exclusion within the stepwise model were set at *P* <.05 and *P* <.10, respectively. All analyses were performed using SPSS, version 26 (IBM).

## Results

Results obtained for the majority of variables verified the Shapiro-Wilk normality test (*P* < .05). However, normal distributions for early flexibility (*P* = .001), late flexibility (*P* = .040), and bilinear torque (*P* = .001) were not demonstrated.

## Foot Type

The mean partial-weightbearing first ray mobility of pooled subjects was 7.2 ± 2.6 mm. Mean partial weightbearing first ray mobility of subjects with a planus foot type was 8.0 ± 2.8 mm compared to 6.0 ± 1.9 mm for those participants in the rectus group, which was a statistically significant difference. Measurements of AHF and partial weightbearing first ray mobility were significantly different across foot type (*P* < .05*)*. The Cohen’s *d* values for these variables were >0.80, indicating that the effect sizes were large. No significant difference in weightbearing first ray mobility was observed. Furthermore, no between-group differences were found for first MTP joint flexibility parameters. Results from the GEE analyses are summarized in Table 3.

**Table 3.** Means, SD, and Results From GEEs for Biomechanical Parameters Across the Planus and Rectus Foot Types.

	Planus (n=23 Feet)		Rectus (n=17 Feet)		GEE Results		Cohen’s d
Parameters	Mean	SD	Mean	SD	χ^2^	*P* Value	Value
AHF (mm/kN)	14.5	1.3	9.3	1.9	**7.578**	.006*	0.83
FRM PWB (mm)	8.0	2.8	6.0	1.9	**8.340**	.004*	0.86
FRM WB (mm)	4.2	2.5	2.9	1.1	3.785	.052	0.65
Early flexibility (degrees/Ncm)	0.47	0.08	0.51	0.06	0.460	.497	0.03
Late flexibility (degrees/Ncm)	0.07	0.01	0.08	0.01	0.924	.336	0.25
Bilinear angle (degrees)	56.0	2.4	63.4	4.7	1.840	.175	0.70
Bilinear torque (Ncm)	184.1	30.4	148.4	15.7	1.222	.269	0.28
Maximum dorsiflexion (degrees)	74.4	2.9	82.9	6.0	1.577	.209	0.66
Laxity (degrees)	47.5	2.0	53.3	4.8	1.166	.280	0.55

Abbreviations: AHF, arch height flexibility; FRM, first ray mobility; GEEs, generalized estimating equations; PWB, partial weightbearing; WB, weightbearing.

* Bold text indicates statistically significant differences (*P* ≤ .05).

## First Ray Hypermobility

**Figure 4.**
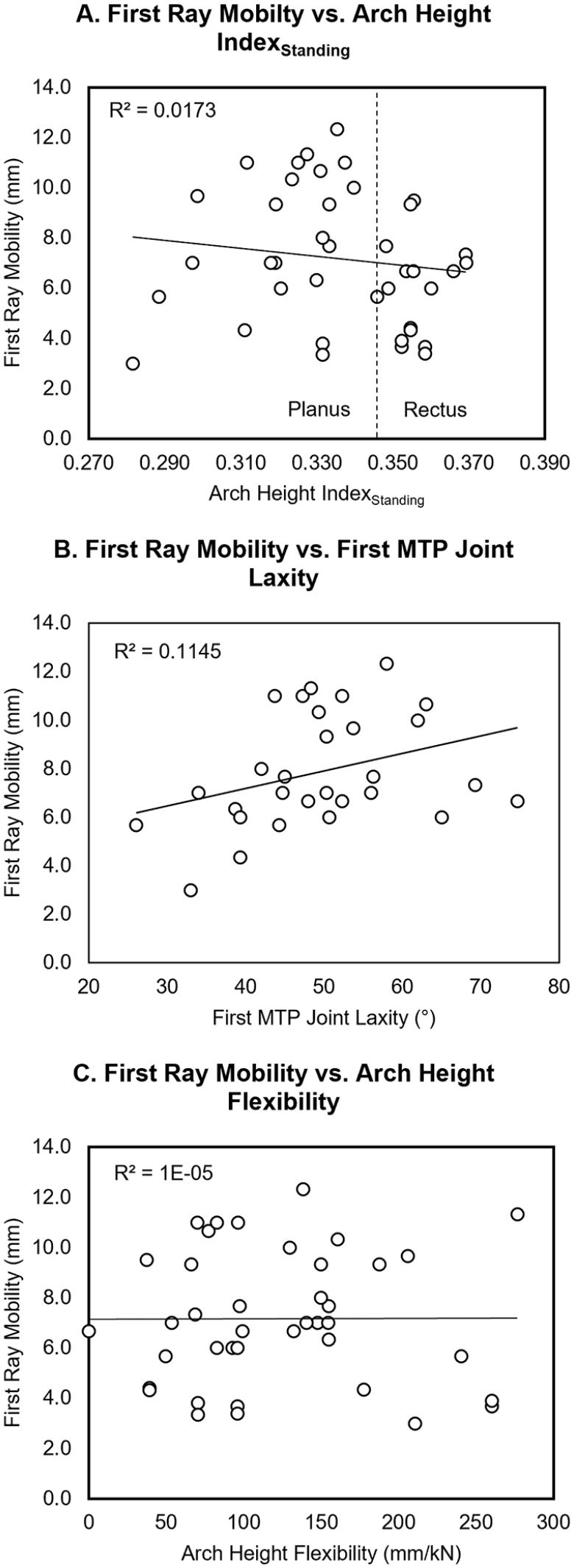
Linear regression analyses of first ray mobility vs (A) AHI_standing_, (B) first metatarsophalangeal joint laxity, and (C) arch height flexibility. AHI, arch height index.

Of the 14 subjects with first ray hypermobility defined as **≥**8 mm, 12 (86%) exhibited a planus foot type. However, 42% (n=11/26) of individuals with <8 mm first ray mobility were also planus in foot type. The hypermobile group exhibited significantly higher (*P* = .000; *d* = 3.54) partial-weightbearing first ray mobility at 10.4 ± 1.2 mm compared with 6.3 ± 1.2 mm for those who were not hypermobile. No other statistically significant differences were observed between the nonhypermobile and hypermobile groups. Furthermore, linear regression analyses found no significant relationships between partial-weightbearing first ray mobility and AHI_standing_ (*R*^2^ = 0.0173, *P* = .418), first MTP joint laxity (*R*^2^ = 0.1145, *P* = .073), and AHF (*R*^2^ = 0.00005, *P* = .983). The linear regression plots are shown in Figure 4. Stepwise linear regression found weightbearing first ray mobility and first MTP joint laxity to be predictive of partial weightbearing first ray mobility, with an R value of 0.613 and *R*^2^ value accounting for 38% of the model variance (Table 4).

**Table 4.** Model Summary From the Stepwise Linear Regression Analyses.

Dependent Variable	Stepwise Linear Regression Model	*R*	*R* ^2^	Adjusted *R*^2^	Significant *F* Change
FRM PWB	FRM WB, first metatarsophalangeal joint laxity	0.613	0.376	0.328	.023*

Abbreviations: FRM, first ray mobility; PWB, partial weightbearing; WB, weightbearing.

*Bold text indicates statistically significant differences (*P* ≤ .05).

## Discussion

The primary finding of this study confirmed the first hypothesis that first ray hypermobility was more common in individuals with a planus foot type, as defined by the AHI_standing_ measurement. In this pilot study, using 8 mm as a cutoff value, 86% of subjects defined by having first ray hypermobility were classified as pes planus.^8,22^ However, 42% of those in the nonhypermobile group were also classified as pes planus, illustrating overlap between these groups. Stepwise linear regression determined weightbearing first ray mobility, and first MTP joint laxity predicted 38% of the variance for first ray mobility.

Previous investigations have found “normal” first ray mobility values ranging from 3 to 8 mm,^1,13-18,21,23,29,36,49^ whereas definitions of first ray hypermobility have ranged between 7 and 10 mm.^13,16,18,23,26,29,36,44,49,51^ In the present study, mean partial weightbearing first ray mobility was 7.2 mm, demonstrating a similar outcome to prior studies^13,23,46^ (Table 5). First ray mobility ≥8 mm acted to discriminate participants with first ray hypermobility in the present work. Consistent with previous descriptions, mean first ray mobility of the nonhypermobile group was within the “normal” range at 6.3 mm. The hypermobile group demonstrated mean first ray mobility of 10.4 mm, which matched a 10-mm threshold proposed by Singh et al.^46^ This between-group difference was statistically significant with a large effect size and may, therefore, provide an objective method of categorizing individuals with first ray hypermobility in future research.

**Table 5.** Means and SD of First Ray Mobility From Previous Research Compared to the Present Study.

Study	Year	Mean Mobility	Load (N)	Method
Jones et al^23^	2005	7.4	N/A	Klaue device
Glasoe et al^15^	2005	6.1	45	Glasoe device
Glasoe et al^16^	2005	5.5	55	Glasoe device
Coughlin and Jones^11^	2007	7.2	N/A	Klaue device
Singh et al^46^	2016	7.2	N/A	Modified Klaue device
Tavara-Vidalon et al^49^	2018	6.5	N/A	Radiographic
Munuera-Martinez et al^36^	2020	6.5	N/A	Handheld ruler
Morgan et al^34^	2021	7.2	50	MAP1^st^

Abbreviation: N/A, not applicable.

Although prior research has postulated an association between planus feet and first ray hypermobility,^22^ few investigations have provided objective measures of foot type alongside first ray mobility.^15,16,23,27,47,49^ Coughlin and Jones^11^ found no significant correlation between increased first ray mobility and arch height. In contrast, Glasoe et al^13^ found that subjects with a valgus forefoot alignment had statistically less (*P* < .05) first ray mobility than subjects with a varus forefoot alignment. Although the current research found no linear relationship between AHI_standing_ and first ray mobility, there was a predominant distribution of individuals with first ray hypermobility who were classified as planus in foot type, confirming the first study hypothesis. This finding agreed with that of Glasoe et al,^13^ where the planus foot may be associated with forefoot varus.^28^ The relationship between foot type and first ray mobility is not fully understood. Despite a predominance of hypermobility among planus individuals, the 42% distribution of subjects who were planus and nonhypermobile indicate factors other than arch height/alignment. Additional research comparing different classification systems of foot type and foot posture^6,27,28,40^ would be useful in understanding these outcomes.

From a clinical perspective, understanding the prevalence of first ray hypermobility by foot type can serve to elucidate potential relationships between foot structure and pathology. It has been suggested that first ray hypermobility may be causative of aberrant function in the planus foot,^1,6,7,22,27,28^ where Hillstrom et al^22^ postulated that a transfer of load from the first to second metatarsal, which occurred in the planus foot during gait, resulted from first ray hypermobility. Furthermore, Menz et al^32^ demonstrated a higher odds ratio of hallux rigidus among individuals with the planus foot type. The first metatarsal of a planus foot, in the presence of first ray hypermobility, may translate excessively in the superior direction, causing the foot to pronate, and redistributing the body’s weight. Once the first ray is at its maximum elevation, the medial band of the plantar fascia may become maximally taut and restrict first MTP joint dorsiflexion during locomotion.^35,44^ As a result of altered structural and functional parameters in the planus foot, excessive loading to the dorsal aspect of the first MTP joint may occur and permit repetitive excessive loading to the articular soft tissues, initiating joint degeneration and leading to hallux rigidus at the end stage.^9,31,32,41,42^

The rotational component of first MTP joint kinematics has typically been defined by dorsiflexion.^1,10,39^ Research in this area has provided conflicting evidence. Buldt et al^7^ reported lower dynamic first MTP joint dorsiflexion in individuals with planus compared to rectus foot types during gait, whereas Rao et al^39^ found no difference with the foot in static weightbearing. Furthermore, Allen et al^1^ compared the first MTP joint dorsiflexion of subjects with “stiff” and “lax” first rays during gait, finding a weak relationship between first ray mobility and first MTP joint dorsiflexion. Similarly, to Allen et al, the present work found a weak relationship between first ray mobility and first MTP joint laxity (*R*^2^ = 0.1145, *P* = .073), rejecting the second study hypothesis. However, the estimated *P* value was close to significant and may have been affected by the study sample size. Rotational laxity of the first MTP joint, which is the amount of angular rotation for a standardized amount of torque applied to the hallux, was measured alongside dorsiflexion. As shown by Roukis et al,^44^ position of the first ray is likely to influence “stiffness” of the first MTP joint during gait, where lowering of the medial longitudinal arch in pes planus may limit the first MTP joint’s mechanical advantage afforded by the Windlass mechanism. However, weightbearing first MTP joint flexibility was not measured in the present study and further investigation with a larger sample size, in the presence of first ray hypermobility, is required to provide objective evidence.

It has been well established that pes planus exhibits a more flexible arch than pes rectus.^22,47,53^ Consistent with previous research, the current study observed significantly more arch height flexibility in subjects who were planus in foot type. As such, flexibility of the arch was expected in hypermobile participants because of dominance of the planus foot type, yet arch height flexibility and first ray mobility were not related, rejecting a relationship between these variables. Although the between-group difference was small and statistically insignificant, it contrasted with conventional belief that flexibility of the medial longitudinal arch may be associated with hypermobility of the first ray. This finding conveys the complexity of foot biomechanics as well as the need for investigation of functional parameters such as plantar loading.

One of the primary limitations associated with this research is the small sample size. The current data may be used for power analysis and act as a basis to design future investigations. Another potential weakness was use of arch height index rather than radiographic criteria for arch type (eg, Meary’s angle). First MTP joint flexibility was not assessed in weightbearing, and though the present information provides a novel understanding of the interaction between flexibility, foot structure, and first ray mobility, analyses in weightbearing would provide a more complete description of first ray mechanics in the presence of hypermobility. The cavus foot type was not included in the current work because of previous findings of this foot type to be protective against foot injuries^24^ and osteoarthritis affecting the midfoot and forefoot.^22,45^ Although participants were asked if they exhibited symptoms of generalized joint hypermobility, as defined by the Beighton criteria,^3^ their general joint mobility was not assessed unless self-reported which may have affected the present results. Although MAP1^st^ may provide reliable assessment of first ray mobility and been indirectly validated with radiographic measurements from the literature, direct validation has not yet been conducted. Finally, bilateral measures of the foot were analyzed as independent samples. Although this may be considered a limitation, GEE was used in the present work to estimate the potential correlation between feet, with correction, by yielding the χ^2^ statistic and corresponding *P* value.

Many structural and functional abnormalities of the first ray have been linked to pes planus, in pursuit of explaining why this foot type has been disproportionately affected by certain orthopaedic conditions.^2,9,31-33,38,42^ The present study of healthy, asymptomatic subjects with planus and rectus foot types established individuals with first ray hypermobility were 86% planus in foot type. Furthermore, an interaction between rotational first MTP joint flexibility and translational first ray mobility was demonstrated using stepwise linear regression. Taken together, these findings provide evidence of first ray hypermobility’s role in aberrant structural parameters of the foot and may act as a basis to investigate the development of symptomatic pathology (ie, collapsing flatfoot, hallux rigidus, and hallux valgus).
